# Evaluation of Stigma Related to Perceived Risk for Coronavirus-19 Transmission Relative to the Other Stigmatized Conditions Opioid Use and Depression

**DOI:** 10.3389/fpsyt.2022.803998

**Published:** 2022-03-11

**Authors:** Sandra Okobi, Cecilia L. Bergeria, Andrew S. Huhn, Kelly E. Dunn

**Affiliations:** School of Medicine, Johns Hopkins Medicine, Baltimore, MD, United States

**Keywords:** COVID-19, opioid use disorder, stigma, opioid, depression

## Abstract

**Background:**

The coronavirus-19 (COVID-19) pandemic was initially characterized by misinformation and fear related to transmission that has been previously shown to produce stigma toward persons perceived to be at risk for transmission. This study evaluated perceptions toward scenarios with variable levels of perceived risk for COVID-19 acquisition, and compared stigma to COVID-19 to depression and opioid use disorder.

**Methods:**

Respondents (*N* = 280) from the United States completed a web-based survey 6 months after pandemic declaration. Questions included demographics and COVID-19 misconceptions, expected response to hypothetical scenarios with variable risk for COVID-19, and the Attribution Questionnaire-9 for COVID-19, depression, and opioid use disorder.

**Results:**

Participants had several COVID-19 misconceptions, including that opioids increased immunity (63.6%), persons were more susceptible based upon racial/ethnic background (63.2%), and underlying health conditions did not influence risk (58.9%). Respondents were highly likely (64/100) to assume someone coughing had COVID-19 and the majority (93.5%) recommended quarantining persons with recent travel. However, the majority of respondents (>70% in all cases) also believed they would not change their COVID-19-related behavior when interacting with persons of different racial, ethnic, and age backgrounds. Finally, persons with COVID-19 engendered greater pity, less fear, less blame, less anger, and more willingness to help from respondents relative to persons with opioid use disorder.

**Conclusion:**

Stigma ratings toward persons perceived at risk of transmitting COVID-19, collected soon after the onset of the pandemic, showed less evidence of stigma relative to persons with opioid use disorder despite pronounced misconceptions regarding COVID-19 risk. Data provide a foundation for additional research in this area.

## Introduction

Severe acute respiratory syndrome coronovirus 2 (SARS-CoV-2), also known as the novel coronavirus-19 (COVID-19), was recognized as a significant health concern in early 2020 and rapidly progressed to become a global pandemic by March 2020 (1). COVID-19 was a new and highly contagious respiratory-based virus, and a massive global effort was undertaken to understand how it was transmitted, the consequences and risk factors for exposure and acquisition of COVID-19, and counter-measures that were needed to mitigate risk and symptom severity. The early stages of discovery were rife with misinformation (2, 3) and substantial fear (4) that was compounded by strong enforcement efforts to slow the spread of the illness and perceived scarcity of existing resources (5).

Notably, the public response to the COVID-19 pandemic had some similarities to what was observed following public recognition of the human immunodeficiency virus (HIV) and other infectious diseases (6–8). Similarities between these illnesses include concerns related to unknown transmission sources, inaccurate perceptions that a specific population of people were responsible for its origin, and lack of information about disease consequences and mitigation strategies, all of which have been found to contribute to stigma and discrimination toward persons perceived to be either responsible or at risk for transmitting the illness. There is now growing evidence that early lack of information and misinformation about COVID-19 also contributed to stigma toward individuals perceived to be responsible or at elevated risk for transmitting COVID-19. This most prominently includes persons of Asian descent, who have experienced substantial levels of discrimination and associated related mental health consequences resultant from COVID-19 (9–11), as well as persons deemed essential workers including first responders, medical personnel, and grocery store attendants (12, 13). COVID-19 related stigma leads to myriad consequences, including unwillingness of individuals to seek treatment that identified themselves as having COVID-19 and significant exacerbations of existing mental health conditions (14, 15).

Stigma, or negative attitudes toward persons as a function of a defining feature, has been shown to produce detrimental effects on the health and well-being of targeted individuals (16, 17). This study was conducted in the early stages of the COVID-19 pandemic, amid growing international reports of stigma being directed toward persons with suspected or confirmed exposure to COVID-19 (18, 19). Given these (at the time only anecdotal) reports, the study aimed to assess the relative stigma that respondents from the United States expressed toward hypothetical persons who displayed behaviors or represented racial/ethnic groups who might be perceived as possessing differential risk for transmitting COVID-19. Moreover, the relative stigma expressed toward persons with COVID-19 was compared to two other highly stigmatized conditions, depression (20, 21) and opioid use disorder (22, 23).

## Materials and Methods

### Participant Recruitment

Respondents were recruited from the crowdsourcing website Amazon Mechanical Turk (AMT) between August 10, 2020 and September 22, 2020. AMT is an online platform that has been recognized as a useful method for disseminating research surveys to broad and representative populations (which was a particularly valuable feature during the COVID-19 stay-at-home orders) (24). The survey was advertised as a “survey on health behaviors” via the mTurk platform and was open to all respondents who were registered on mTurk, were over the age of 18, and resided in the United States. Survey questions were hosted on Qualtrics (Provo, UT, United States). Interested individuals completed an eligibility screen to determine initial study eligibility and were informed that their participation was voluntary and that completing the study served as their consent to participate. Respondents were compensated 0.10 for competing the eligibility survey and $3 for survey completion. The Johns Hopkins University IRB acknowledged this study and the survey methods conformed to the Checklist for Reporting Results of Internet E-Surveys (CHERRIES) (25).

A total of 316 respondents were eligible and completed the survey. Among them, data from 36 persons (11.4% of those who began the survey) were removed due to inaccurate responding on one of several embedded quality control attention checks distributed throughout the survey. The final analyzed convenience sample was *N* = 280 (88.6% of those eligible for the survey).

### Measures

#### Demographics and Characterization of Coronavirus-19 Knowledge

Respondents were asked standard demographic questions to characterize the sample, and whether they had ever tested positive for COVID-19. Awareness of prominent facts and myths associated with COVID-19 was then assessed by asking respondents to indicate their opinion on the following unconfirmed messages being shared by persons in a hypothetical group chat setting (1) “people with an Asian background are the “spreaders” of the virus,” (2) people who do not wear masks in public are more susceptible to contracting and spreading the virus,” (3) “extreme heat can kill the virus,” (4) “only individuals of certain races or ethnicities can be infected,” (5) “people with underlying health conditions are not at an increased risk of contracting the virus,” and (6) “individuals who take opioids are immune.” Message order presentation was randomized and respondents rated their degree of agreement on an 8-point Likert scale (strongly agree to strongly disagree).

#### Perceptions as a Function of Coronavirus-19

Given the lack of existing questions related to COVID-19 stigma, a series of scenarios meant to reflect real-world decision points were developed to assess general attitudes toward persons who may be suspected of having COVID-19 or being recently exposed either on the basis of their behavior or as a function of their race/ethnicity using the following questions.

Attitudes based upon behavior were assessed via a four-item block that asked the respondent to rate their reaction to four scenarios. The first two scenarios asked them to rate their perceived likelihood a friend of theirs may have COVID-19 on a 0 (not at all) to 100 (extremely) scale when they observed the individual (1) coughing or (2) displaying flu-like symptoms. These questions demonstrated acceptable internal validity (Chronbach’s alpha = 0.72). The second two scenarios asked participants whether they thought an individual should complete a 14-day quarantine after returning from a state with high levels of COVID-19 and (3) did display symptoms of COVID-19 or (4) did not display symptoms of COVID-19 should be tested for COVID-19. Questions were rated using a four-item ordinal scale (definitely yes, probably yes, probably no, definitely no) and presentation order was randomized within the four-item block. These items demonstrated poor internal reliability (Chronbach’s alpha = 0.40).

Attitudes based upon race and ethnicity as they pertained to risk for COVID-19 exposure were measured by presenting respondents with four new scenarios in which persons of different racial backgrounds (Asian–American, Caucasian, African–American) were wearing a mask and approaching them in a grocery store aisle. Since age had been publicized as being associated with differential risk for acquiring a severe form of COVID-19, two additional scenarios were added that held ethnicity constant as Hispanic and varied the age of the individual (e.g., elderly, young person). For each of these five conditions, respondents were asked to select one of the following behavioral response options: (1) continue shopping, (2) continue shopping while maintaining 6 feet distance, (3) leave the aisle. Order of question presentation was randomized within this block. These questions demonstrated strong internal reliability (Chronbach’s alpha = 0.82).

The perceived risk for acquiring COVID-19 for persons with compromising health conditions was assessed by asking respondents to envision themselves as (1) a middle-aged individual with no known compromising health condition and then (2) a middle-aged individual with a known compromising health condition. Respondents were then asked how safe they would feel around their (1) friend, (2) co-worker, and (3) family member if that individual was visiting them after self-quarantining for 14-days and re-testing negative for COVID-19. Responses were rated on a scale of 0 (extremely unsafe) to 100 (extremely safe) and order of question presentation was randomized within this block. These questions demonstrated strong internal reliability (Chronbach’s alpha = 0.94).

#### Attribution Questionnaire-9

The Attribution Questionnaire-9 (AQ-9) (26) is a nine-item measure that assesses public stigma toward individuals with mental illnesses. Respondents completed the measure three times in response to three different framing contexts: an individual (1) who had depression and who has been unable to get out of bed or shower for several days and was recently hospitalized for their symptoms (Chronbach’s alpha = 0.84); (2) who had opioid use disorder and had been experiencing opioid withdrawals for several days and was recently hospitalized for their symptoms (Chronbach’s alpha = 0.82); and (3) who was an essential employee working at a major supermarket who had a pre-existing health condition and had been experiencing dry cough, loss-of-taste, and running a fever for several days and was recently hospitalized for their symptoms (Chronbach’s alpha = 0.80). Response options required respondents to indicate whether they felt: (1) (pity) pity for the individual, (2) (danger) that the individual was dangerous, (3) (fear) scared of the individual, (4) (blame) that the individual was to blame for their present condition, (5) (segregation) that the individual should enter a treatment center, (6) anger toward the individual, and (7) (help) they would help the individual, on a 1 (not at all) to 7 (very much) scale. AQ-9 questions related to the domains of “coercion” and “avoidance” were not collected due to an error in the survey delivery program. Each item from the AQ-9 represents a unique factor and serves as its own primary outcome. The order in which question blocks were presented (depression, opioid, COVID-19) was randomized and all respondents completed all blocks.

### Data Analysis

Respondent demographics and responses to COVID-19 misperceptions were summarized descriptively and presented in Table 1. Binary logistic regressions were used to assess whether the racial/ethnic background of an individual significantly impacted participant willingness to continue shopping in a grocery-store aisle with that individual. Paired *t*-tests were used to compare the degree to which a compromising condition was perceived to modify risk for acquiring COVID-19. Individual ASQ-9 ratings were evaluated using one-way (condition) repeated measures analysis of variance (ANOVA); effect size estimates are presented as partial eta squared (>0.1 small effect, >0.6 medium effect, >0.14 large effect). A power analysis that was conducted assuming repeated measures analyses of within-subject effects across three items using a single administration and setting alpha to 0.05 determined that a sample size of 43 provided 95% power to detect a main effect of condition. Alpha was set at 0.05 and all analyses were conducted using SPSS v. 15.

**TABLE 1:** Respondent characteristics.

Male (%, *n*)	68.2 (191)
Age in years (mean, SD)	36.7 (10.2)
**Self-described Residential Location (%, *n*)**	
Urban	56.7 (159)
Suburban	30.4 (85)
Rural	12.8 (36)
**Highest level of education (%, *n*)**	
High school or lower	3.9 (11)
Some college	6.4 (18)
2 or 4 year college degree	65.4 (183)
Masters or terminal degree	23.9 (67)
Working full or part-time past 30 days (%, *n*)	92.9 (260)
**How knowledgeable are you about COVID-19? (%, *n*)**	
Extremely	29.2 (82)
Very	41.1 (115)
Moderately	26.1 (73)
Slightly	3.6 (10)
Not	0
Essential employee (%, *n*)	71.4 (200)
Tested positive for COVID-19 (%, *n*)	28.6 (80)
Been around someone who tested positive for COVID-19 (%, *n*)	57.9 (162)

## Results

### Respondents

Respondents (*N* = 280) were 37 (SD = 10) years old, had completed some college (96%), and had been employed full or part time in the past 30 days (93%), with 71% of those individuals indicating they were considered essential workers. All participants considered themselves to be at least slightly knowledgeable about COVID-19 and 47% had been tested for COVID-19, with 33% of participants receiving a positive diagnosis (see Table 1).

Although participants felt they were well-informed about COVID-19, evaluation of specific information related to the virus revealed important knowledge gaps. For instance, 63.6% of participants were either uncertain or believed that opioid medications could increase immunity to the virus, 63.2% believed only individuals of specific racial or ethnic backgrounds could acquire COVID-19, 58.9% did not believe underlying health conditions meaningfully changed risk of acquiring COVID-19, 56.4% believed that persons of Asian descent were “spreaders” of the virus, and 50% felt extreme heat could kill the virus. Only one item, that use of masks to decrease virus susceptibility, was answered correctly by the majority (73.9%) of participants.

### Attitudes Based Upon Known Behavior

On a scale of 0 (not at all) to 100 (extremely), participants indicated they were somewhat likely to assume a friend had COVID-19 if the individual was heard coughing (64.1, SD = 25.1) and confidence in this rating increased if the individual described having flu-like symptoms (71.1, SD = 20.5). Upon learning that an individual had recently flown from an area with a high COVID-19 positivity rate, the majority (93.5%) of participants indicated the individual should quarantine for 14-days if he or she was not showing symptoms of COVID-19, and 77.5% believed the individual should quarantine even if he or she was not showing symptoms of COVID-19.

Varying the race and ethnicity of another shopper in a grocery store aisle did not have a major impact on whether participants would continue to shop in that aisle. Specifically, the vast majority of participants indicated they would continue shopping in an aisle with other individuals if they could remain at a 6-foot distance, regardless of whether the shopper was of Asian descent (77.5%), Caucasian (72.5%), African American (72.1%), elderly (71.4%), or a child (75%). Only 3.6% - 6.8% of participants indicated they would leave the aisle altogether. Binary logistic regression was not significant (*p* = 0.612), indicating the described racial or ethnic background of the target individual did not significantly impact participant’s reported willingness to continue shopping.

Evaluation of perceived COVID-19 risk as a function of underlying health condition revealed that in all cases, an individual with COVID-19 was perceived as a greater risk to someone who had an underlying condition even when that individual had tested negative for COVID-19. Specifically, risk to persons with and without underlying conditions (on a 0–100 scale) was perceived as less safe even after a negative COVID-19 test with regard to a friend visiting their house [61.2 (22.5) vs. 65.2 (24.9), *t*(279) = 3.6, *p* < 0.001], a coworker returning to work [62.7 (25.3) vs. 66.8 (23.2), *t*(279) = 3.67, *p* < 0.001], and a family member returning home [64.9 (25.5) vs. 67.9 (23.1), *t*(279) = 2.5, *p* = 0.12].

### Comparison of Coronavirus-19 to Other Stigmatized Conditions

Table 2 provides results from a repeated measures analysis of the ASQ-9 items. Data revealed a main effect of condition, whereby persons identified as having COVID-19 generally had less stigma directed toward them relative to persons who had depression and then opioid use disorder (in that order). Specifically, persons identified as having COVID-19 received higher levels of pity, lower feelings of fear, less blame for their condition, less anger, and more willingness to help as compared to persons with opioid use disorder. Despite these differences, participants were largely positive toward persons with opioid use disorder as well, stating they were fairly likely to help the individual and felt a moderate level of pity toward the person. However, evaluation of effect sizes suggests that, despite reaching significance, differences were all in the low effect size with the exception of the “blame” item that achieved moderate effect size.

**TABLE 2:** Stigma ratings.

	COVID-19	OUD	Depression		
				
	Mean SD	Mean SD	Mean SD	*F* (df), *P*-value	Effect Size
**ASQ-9 Dimensions (range 1 none at all-7 very much)**					
Pity: I would feel pity for the individual	5.4^a^ ± 1.4	5.3^b^ ± 1.4	5.5^c^ ± 1.3	*F* (2,2798) = 30.8, *p* < 0.001	0.02
Dangerousness: How dangerous is the individual	4.6^a^ ± 2	4.5 ± 1.7	4.7^b^ ± 1.7	*F* (2,2798) = 31.7, *p* < 0.001	0.02
Fear: How scared of the individual would you feel	4.4^a^ ± 2	4.7^b^ ± 1.8	4.8^c^ ± 1.6	*F* (2,2798) = 43.3, *p* < 0.001	0.03
Blame: I think it is the individual’s own fault that he/she is in this condition	4.2^a^ ± 1.9	4.9^b^ ± 1.6	4.3^c^ ± 1.9	*F* (2,2798) = 128.2, *p* < 0.001	0.08
Segregation: I think it would be best for the individual to be put in a treatment center	4.6a ± 1.8	5.0^b, c^ ± 1.5	4.9^b,d^ ± 1.8	*F* (2,2798) = 54.7, *p* < 0.001	0.04
Anger: How angry do you feel toward the individual	4.0^a^ ± 2.1	4.2^b,c^ ± 1.8	4.0^d^ ± 2	*F* (2,2798) = 23.8, *p* < 0.001	0.02
Help: How likely is it that you would help the individual	5.4 ± 1.5	5.2 ± 1.5	5.3 ± 1.5	*F* (2,2798) = 13.4, *p* < 0.001	0.01

*Different subscript letters denote significant differences. ASQ-9, Attribution Questionnaire; OUD, opioid use disorder; SD, standard deviation; df, degrees of freedom. Partial eta square > 0.01 = small, >0.06 = moderate, >0.14 = large.*

## Discussion

This study assessed perceptions of persons from the general public related to risk for acquiring COVID-19 and its relationship toward perceived stigma related to COVID-19. COVID-19 associated stigma was compared to stigma ratings related to depression and opioid use disorder, two conditions with documented high levels of public stigma (20–23). Data were collected approximately 6 months after COVID-19 was declared a global pandemic, which provided ample time for respondents to have been exposed to both accurate and misinformation. Results revealed the majority of respondents believed several important misconceptions regarding COVID-19 transmission risks to be true. Nevertheless, respondents demonstrated relatively low levels of stigma toward persons based upon their perceived potential for transmitting COVID-19, their racial/ethnic backgrounds, and their known risk behaviors. When directly compared to depression and opioid use disorder, COVID-19 engendered the lowest ratings of stigma, whereas opioid use disorder engendered the highest ratings.

This sample of US-based respondents showed some evidence of stigma toward persons of Asian descent (through their endorsement that this group was a prominent spreader of COVID-19) but outwardly believed that race and ethnicity would not impact how they behaved around persons of various racial and ethnic backgrounds (evident in their expected behavior while grocery shopping). The inaccurate understanding regarding the origin of COVID-19 contagion is discouraging, especially in the context of increased discrimination and violence toward persons of Asian descent following the COVID-19 outbreak (9–11). These reports are likely associated with a recognized increase in social media channel reports that endorsed and propagated the erroneous notion that persons of Asian descent were responsible for transmitting COVID-19 (3, 27). The recent increases in violence observed toward persons of Asian descent suggest these data may have been an early signal of public attitudes on this issue. This effect is also evident in other countries, in which profound stigma toward persons perceived to be at elevated risk for transmitting COVID-19 has been observed (28, 29).

These data also add to a growing literature examining how health perception related to COVID-19 may influence COVID-related risks and consequences. In this study, respondents generally felt they were well-informed about COVID-19, yet their responses revealed profound gaps in understanding that could increase their risk for acquiring COVID-19 and also influence their impression of persons who tested positive for COVID-19. This included beliefs such as opioid medications influencing vulnerability for the virus, that health comorbidities did not contribute to virus acquisition, and that race and/or ethnicity increased the risk profile for viral transmission. The fact that three quarters of respondents were essential workers suggests they may have felt highly vulnerable to COVID-19, which has been associated with having a poor psychological response to COVID-19 information (30). This is further supported by evidence that being misinformed about the COVID-19 virus was more closely associated with personality traits such as low empathy and self-efficacy than demographic-level characteristics (31). It is also the case that the high levels of misinformation held by respondents could have buffered them against mental health consequences of the pandemic. The degree to which this occurred, as well as the origins of the misinformation here (32), were not queried and remain unknown. However, this type of misinformation has real-world consequences; inaccurate beliefs related to COVID-19 risks and consequences correspond to reduced willingness to become vaccinated against the virus (33) and persons who were overconfident in their misinformation showed elevated risk for developing mental health-related consequences during the pandemic (34). Thus, the data collected here suggest that the inaccurate beliefs held by large percentages of respondents had the potential to be detrimental to both them and the persons around them. Considerable advancements in COVID-19 knowledge have been made since these data were collected and it is important that these concepts be reassessed to see whether these inaccurate beliefs persisted. Interventional efforts to address misinformation and promote pro-health behaviors should also be considered.

Stigma ratings toward hypothetical persons with depression and opioid use disorder were used as positive controls for this study based upon documented public stigma toward these conditions. Consistent with prior research (35), when compared to other medical and mental health conditions, opioid use disorder remained the most stigmatized condition. Stigma toward persons with opioid use disorder is a known issue that prevents patients from initiating treatments and communities from expanding treatment access (36, 37). This is significant in the context of COVID-19, during which rates of overdose from opioids have continued to accelerate at unprecedented rates (38–40). The fact that this effect was found both serves as a positive control that strengthens the results of the study and also reiterates the need for stigma-mitigation strategies for opioid use disorder.

The fear and misinformation present during the onset of COVID-19 was highly reminiscent of the societal response to HIV in the 1980’s and may serve as a model for addressing concerns regarding COVID-19 (7, 41). Stigma toward persons with HIV has now decreased substantially (42), coincident with improved knowledge regarding acquisition risks and protections, development of effective treatments, and major public health campaigns to directly combat HIV misinformation. Efforts are already underway to combat stigma related to COVID-19 (2, 43), though our data suggest that stigma toward persons who acquired COVID-19 specifically may be lower than what is observed for other transmissible conditions.

This study was intentionally brief for data collection purposes though the brevity precludes more in-depth examinations of relationships between COVID-19 exposure and knowledge with stigma ratings. Due to the lack of precedent for COVID-19 specific scales, the study developed questions to assess attitudes toward COVID-19 and results could have been impacted by phrasing in ways that cannot be determined. Despite achieving significance, the effect sizes for the majority of AS-9 comparisons were low, suggesting that the clinical relevance of these differences may be minimal. Finally, information about COVID-19 continues to change rapidly and it is likely that the attitudes and/or knowledge we collected pertaining to COVID-19 have continued to evolve. Nevertheless, this study provides an initial glimpse at stigma directed toward persons as a function of COVID-19 and suggests respondents attributed relatively low levels of stigma to individuals perceived to be at high risk for COVID-19 transmission, though stigma toward persons with depression and especially opioid use disorder remained evident. These data can be used to support more focused examination of stigma and related consequences in response to medical and other chronic conditions.

## Data Availability Statement

The raw data supporting the conclusions of this article will be made available by the authors, without undue reservation.

## Ethics Statement

The studies involving human participants were reviewed and approved by Johns Hopkins IRB. Written informed consent for participation was not required for this study in accordance with the national legislation and the institutional requirements.

## Author Contributions

SO, CB, and KD developed and conducted the study. All authors contributed to the data analyses, interpretation, manuscript preparation, and approved the submitted version.

## Conflict of Interest

In the past 3 years KD has consulted for Canopy Corporation, Beckley-Canopy, and MindMed. The remaining authors declare that the research was conducted in the absence of any commercial or financial relationships that could be construed as a potential conflict of interest.

## Publisher’s Note

All claims expressed in this article are solely those of the authors and do not necessarily represent those of their affiliated organizations, or those of the publisher, the editors and the reviewers. Any product that may be evaluated in this article, or claim that may be made by its manufacturer, is not guaranteed or endorsed by the publisher.
